# Supplementary material for: Assessment of Stakeholder’s Perceptions of the Value of Coral Reef Ecosystem Services: The Case of Gili Matra Marine Tourism Park
Source: Int J Environ Res Public Health. 2022 Dec 21;20(1):89. doi: 10.3390/ijerph20010089 (PMC9819916; doi:10.3390/ijerph20010089)
Supplement: Supplementary file 1 [file ijerph-20-00089-s001.zip › ijerph-1919399-supplementary.pdf]

Supplementary Materials S1

STAKEHOLDER VALUATION OF THE GMMTP'S CORAL REEF ECOSYSTEM SERVICES
INTERVIEW SHEET

The interview aims to assess the ecosystem services supply and the stakeholders' perception of the ecosystem services. The stakeholder's perception will be based on participant's assigned values, which includes the importance and vulnerability of the ecosystem services, magnitude of their dependency, and associated preference upon the ecosystem services. This interview guide will not be used as a formal schedule of questions and to be followed word-to-word. This guide is aimed to use for the research team to cover all courses of the interview. The research team may:

- Ask questions not indicated in this interview guide to generate follow-up responses.
- Follow through a new course of discussion when new interesting issues emerged.
- Be expected to ask for an example for the majority of the questions.
- Be expected to take note of the gestures and expressions of the interviewee.

The research team is required to provide and educate the participants regarding the participant information sheet and participant consent form before the start of the interview.

Background information

- Background, role, and responsibility of the interviewee.

General information of the ecosystem services

- Do these ecosystem services exist within the Gili Matra area?
- Where does these ecosystem services found within the Gili Matra area?

Please indicate your perception on the importance of the following ecosystem services to human well-being
(1= not at all important; 2=low importance; 3=neutral; 4=important; 5=very important)

- Provisioning services
    - Xx (following the result of ES identification & mapping)
- Cultural services
    - Xx (following the result of ES identification & mapping)
- Regulating services
    - Xx (following the result of ES identification & mapping)
- Supporting services
    - Xx (following the result of ES identification & mapping)

Please indicate your perception on the level of vulnerability of the following ecosystem services to any forms of threats or stressors that may result in the degradation or loss of the services
(1= not at all vulnerable; 2=low vulnerability; 3=neutral; 4=vulnerable; 5=highly vulnerable)
Provisioning services

- Xx (following the result of ES identification & mapping)
- Cultural services

    - Xx (following the result of ES identification & mapping)
- Regulating services
    - Xx (following the result of ES identification & mapping)
- Supporting services
    - Xx (following the result of ES identification & mapping)

Please indicate your level of dependency to the following ecosystem services
Note: dependency refers to the extent in which the ES assists in their daily lives and livelihood
(1= not dependant; 2=slightly dependant; 3=neutral; 4=dependant; 5=highly dependant)
Provisioning services
    - Xx (following the result of ES identification & mapping)
- Cultural services
    - Xx (following the result of ES identification & mapping)
- Regulating services
    - Xx (following the result of ES identification & mapping)
- Supporting services
    - Xx (following the result of ES identification & mapping)

Please indicate your level of preference to the following ecosystem services for further management and/or intervention priority
(1= no priority; 2=low priority; 3=neutral; 4=priority; 5=highest priority)
- Provisioning services
    - Xx (following the result of ES identification & mapping)
- Cultural services
    - Xx (following the result of ES identification & mapping)
- Regulating services
    - Xx (following the result of ES identification & mapping)
- Supporting services
    - Xx (following the result of ES identification & mapping)

Additional questions:

- What is the level of stakeholders' influence on the ecosystem services?
- Who has direct or indirect impact on the ecosystem services?
- How would the stakeholders' activities be affected if all is conducted simultaneously?
- What is the stakeholders willingness and capacity to participate in the implementation of management regulation?
- What are the current and future interests of the various stakeholders in the use and management of the ecosystem services?
- What are the current and future expectations of the various stakeholders in the use and management of the ecosystem services?
- What are the social and environmental impacts, both positive and negative, of their past and current uses of and relationships with the ecosystem services?

-End of interview-

References:

Aaltonen, K. (2011). Project stakeholder analysis as an environmental interpretation process. *International Journal of Project Management*, *29*(2), 165–183. https://doi.org/10.1016/j.ijproman.2010.02.001

Aziz, A. A., Thomas, S., Dargusch, P., & Phinn, S. (2016). Assessing the potential of REDD+ in a production mangrove forest in Malaysia using stakeholder analysis and ecosystem services mapping. *Marine Policy*, *74*, 6–17. https://doi.org/10.1016/j.marpol.2016.09.013

Supplementary Materials S2

Expert's Assessment on Key Stakeholder Groups related to Coral Reef Ecosystem Services in Gili Matra Marine Tourism Park (GMMTP)

Research Information

The objective of this research is the identification and valuation of existing ecosystem services in the coral reef clusters of TWP Gili Matra. The valuation of ecosystem services is assessed through the perceptions of stakeholders who interact with ecosystem services on a daily basis. This survey is part of a study that aims to identify the value of the closeness of stakeholder groups to coral reef ecosystems in TWP Gili Matra. Thank you for your participation in this survey. Data recorded in this survey will be protected under the University of Queensland's research code of conduct.

1. How familiar are you with the stakeholder groups related to the coral reef ecosystem in the GMMTP area?
   - ☐ Not familiar (no knowledge at all)
   - ☐ Familiar (knows in general but not in detail and has never interact with)
   - ☐ Very familiar (knows most of the stakeholders and interacts with quite often)

2. Please provide your assessment on the relevance* of the stakeholder groups below to the coral reef ecosystems in TWP Gili Matra.
   **relevance refers to the closeness/intensity of interaction between stakeholder groups and coral reef ecosystems*

No	Stakeholder Group	1 (Not relevant)	2 (Less relevant)	3 (Relevant)	4 (Very relevant)
1	Dept. of Tourism				
2	Dept. of Forestry				
3	BKKPN Kupang				
4	Dept. of Fisheries				
5	Village Government				
6	Dive Centre				
7	Snorkelling & Glass bottom boat operator				
8	Tourism Community				
9	Conservation Community				
10	Surveillance Community				
11	Fishermen Community				
12	NGO				

13	Youth Community				
14	Women Group				
15	Hotel Operators and Restaurants				
16	Cart Rental and Bicycle				
17	District Government				
18	Higher Education				

3. Please provide your assessment of the level of influence* of the stakeholder groups below to the coral reef ecosystem in TWP Gili Matra.
**influence refers to the magnitude of the impact (positive or negative) of stakeholder activities/decisions/interventions on coral reef ecosystems*

No	Stakeholder Group	1 (No influence)	2 (Less Influence)	3 (Influential)	4 (Very Influential)
1	Dept. of Tourism				
2	Dept. of Forestry				
3	BKKPN Kupang				
4	Dept. of Fisheries				
5	Village Government				
6	Dive Centre				
7	Snorkelling & Glass bottom boat operator				
8	Tourism Community				
9	Conservation Community				
10	Surveillance Community				
11	Fishermen Community				
12	NGO				
13	Youth Community				
14	Women Group				
15	Hotel Operators and Restaurants				
16	Cart Rental and Bicycle				
17	District Government				
18	Higher Education				

4. Do you have input regarding stakeholder groups that are not included in the list above? Please provide your input below along with its relevance and impact rating.

Format: (Group), (relevance score), (influence score); Example: 1. Aquaculture group, 3, 2.

Skip the question if you have no input/ comments.

5. How confident are you in your estimation/assessment of the relevance and influence of stakeholder groups on the coral reef ecosystems in GMMTP?

- ☐ Not confident at all
- ☐ A little confident
- ☐ Confident
- ☐ Very confident

-End of assessment-
